# Is there a risk of developmental problems in infants born to mothers with gestational diabetes mellitus and/or pre-eclampsia?

**DOI:** 10.1371/journal.pone.0318003

**Published:** 2025-01-24

**Authors:** Bilge Nur Yardımcı-Lokmanoğlu, Yusuf Topal, Esra Kınacı-Biber, Zeynep Arıkan, Gülsen Sırtbaş-Işık, Doğan Porsnok, Hasan Tolga Çelik, Akmer Mutlu

**Affiliations:** 1 Developmental and Early Physiotherapy Unit, Faculty of Physical Therapy and Rehabilitation, Hacettepe University, Ankara, Türkiye; 2 School of Physical Therapy and Rehabilitation, Ahi Evran University, Kırşehir, Türkiye; 3 Department of Physiotherapy and Rehabilitation, Faculty of Health Sciences, Karamanoğlu Mehmetbey University, Karaman, Türkiye; 4 Department of Physiotherapy and Rehabilitation, Faculty of Health Sciences, Yüzüncü Yıl University, Van, Türkiye; 5 Faculty of Physical Therapy and Rehabilitation, Bingöl University, Bingöl, Türkiye; 6 Neonatology Unit, Department of Child Health and Diseases, Faculty of Medicine, Hacettepe University, Ankara, Türkiye; University of Gondar, ETHIOPIA

## Abstract

**Objective:**

The aims of this study were (i) to describe the early spontaneous movements in 3-to 5-month-old infants in groups of infants born to mothers with GDM and/or PE, (ii) to compare them, and (iii) to analyze the differences between infants with these risk factors and typically developing infants born to mothers without GDM and/or PE and other risk factors.

**Methods:**

This cohort study included 255 infants in 4 groups: (i) 96 infants born to mothers with GDM, (ii) 78 infants born to mothers with PE, (iii) 31 infants born to mothers with GDM and PE, and (iv) 50 typically developing infants. Early spontaneous movements, including not only fidgety movements but also concurrent movement and postural patterns, were assessed using the General Movements Assessment (GMA), which determines the Motor Optimality Score-Revised (MOS-R). Comparisons were made using one-way ANOVA for normally distributed continuous variables, Kruskal-Wallis test for non-normally distributed data, and Pearson chi-squared for categorical variables. Univariate logistic regression analyses were used to determine the odds ratios (OR) with 95% confidence intervals (CI).

**Results:**

There were no differences between the three groups, which included infants whose mothers had at least one of these risk factors (p>0.05). Infants born to mothers with GDM, infants born to mothers with PE, and infants born to mothers with both GDM and PE had more aberrant fidgety movements, reduced age-adequate movement repertoire, and more abnormal postural patterns than typically developing infants (p<0.05), in addition to lower MOS-R. When looking at those with ≤24 in MOS-R, the odds ratios were ≥2.74.

**Conclusion:**

Findings suggest that early spontaneous movements, GMA, may play a crucial role in understanding developmental outcomes of these infants and in determining infants who need early intervention.

## Introduction

Gestational diabetes mellitus (GDM) and pre-eclampsia (PE) are associated with an increased risk of complications and negative effects on infants’ health [1, 2]. GDM is characterized by diabetes and carbohydrate intolerance with the onset of pregnancy [3]. A recent systematic review and meta‑analysis reported that the prevalence of GDM was 10.6% worldwide [3]. PE is another disorder in pregnancy and is characterized by onset hypertension and proteinuria after 20 weeks of gestation [4]. It is estimated to affect 4.6% of pregnancies [5]. The prevalence of GDM and PE in our country, Türkiye, are 7.7% and 7.2, respectively [6, 7]. Various previous studies focused on the association between both risk factors [8–10], and some authors revealed that GDM is a risk factor for PE [8, 9]. Recently, Yang et al., indicated that many studies have investigated the effect of GDM on the predisposition to PE [11]. They are becoming more common, and it has been stated that they can be considered as important global public health concerns [12, 13].

The newborns born to mothers with GDM might be observed fetal hyperinsulinemia, which results in macrosomia: a risk factor for asphyxia and perinatal death, and shoulder dystocia and nerve injuries, neonatal hypoglycemia (low blood glucose), hyperbilirubinemia (high bilirubin levels) [14, 15]. PE has also increased the risk of preterm birth and intrauterine growth restriction (IUGR) [2]. In addition to these complications seen in the early period, some long-term studies have also shown that there are different developmental delays in the motor, intellectual, or language domain, in children born to mothers with GDM or PE [16–19]. He et al. [18] have reported that children born to mothers with GDM were at risk of motor developmental delays at 12 months old, and Ornoy et al. [20] also had similar findings on fine and gross motor development at 5–8 years of age. Infants born to mothers with PE before 32 weeks of gestation had a lower score on motor development at the age of 2 years [19]. Some studies have also shown that children born to mothers with GDM or PE might have cognitive and language delays [16, 19].

Furthermore, there might be changes in the brains of the infants born to mothers with GDM or PE [21–25]. It has been reported that infants born to mothers with GDM had white matter abnormalities in different brain regions [21], and altered volumes of left inferior hippocampal body [23]. Linder et al. [24] also revealed that infants born to mothers with GDM had slower postprandial brain responses in the fetal period, and this could have affected fetal brain development. Recently, Xing et al. [25] found that PE might cause brain development delay in the posterior limbs of internal capsule, superior frontal gyrus, and splenium of the corpus callosum. Additionally, altered volumes of the cerebellum, temporal lobe, brain stem, and reduced cerebral vessel radii were shown in infants born to mothers with PE [22].

Early detection of long-term problems and of integrity of the developing nervous system has gained much attention, infants could be predicted for risk of neurologic disorder, especially cerebral palsy (CP), before 5 months of age using general movements assessment (GMA) [26]. On the other hand, there are also efforts to define fidgety movements in diseases such as various genetic syndromes other than CP [27–29]. Detailed GMA is included fidgety movements and other movement and postural patterns in infants at 3-to 5-months post-term age, and motor optimality score (MOS) is also determined [30, 31]. Abnormal general movements (GMs) were related to abnormal white matter in very preterm infants [32, 33], basal ganglia and thalami lesions in term infants who had perinatal asphyxia [34], and reduced cerebellar diameter in very preterm infants [35]. The MOS was related to motor performance in children born with very low birth weight [36], cognitive development in children born preterm [37], and language performance in typical children [38]. The MOS was also found to be lower in infants with hypoxic-ischemic encephalopathy (HIE) [39], while motor optimality score-revised (MOS-R) was lower in term-infants with hyperbilirubinemia compared to the control group [40]. Considering the high predictive value of GMs in neurological disorders and the relationship of MOS (or MOS-R) with developmental parameters [26, 36–38], we hypothesize that GMA may also be affected in relation to brain effects and delays or risks in developmental domains in infants born to mothers with GDM and PE. To the best of our knowledge, infants born to mothers with GDM and/or PE have not been examined in this earliest period, at 3-to 5-months of age, using detailed GMA in different groups, although GDM and PE are known to affect infant development or brain and are associated with each other.

The aims of this study were: (i) to investigate the detailed GMA results in 3- to 5-month-olds in three groups; including infants born to mothers with GDM (group 1), infants born to mothers with PE (group 2), and infants born to mothers with GDM and PE (group 3), (ii) to compare infants’ MOS-R and its subcategories between all three groups, and (iii) to analyze the differences in the MOS-R and its subcategories in three groups and the control group (group 4) who had a normal neurological outcome at 3–5 years of age.

## Material and methods

### Study design

This retrospective cohort study included 255 infants, 205 infants born to mothers with GDM and/or PE and 50 infants in the control group who were referred between 01 January 2015–01 October 2021.

### Study setting and data source

All at-risk infants referred from the Division of Neonatology to the Faculty of Physical Therapy and Rehabilitation, Developmental and Early Physiotherapy Unit, Hacettepe University are routinely evaluated and followed up under the same conditions from 2015. The referred infants at risk are followed up with age-appropriate evaluation methods, one of which is the GMA before the 20 weeks of age. The infants were divided into three groups: (group 1: n = 96) infants born to mothers with GDM, (group 2: n = 78) infants born to mothers with PE, and (group 3: n = 31) infants born to mothers with GDM and PE. Exclusion criteria were congenital malformations, or genetic syndromes. Table 1 shows the clinical characteristics of the infants, and maternal characteristics are given in Table 2. In addition, 50 infants born term to mothers without GDM and/or PE, having no other risk factors, and normal neurological outcome at 3–5 years of age, were included as a control group for comparison from our GMA database (group 4). Post-hoc power calculation for the differences in MOS between all groups showed that the study had 95% power using G*Power 3.1. Ethical permission for this retrospective study was provided by the Non-interventional Clinical Research Ethics Board, Hacettepe University with a waiver of informed consent (GO 21/1066).

**Table 1:** Clinical characteristics of all infants (n = 255).

Variables		Group 1 Infants born to mothers with GDM (n = 96)	Group 2 Infants born to mothers with PE (n = 78)	Group 3 Infants born to mothers with GDM and PE (n = 31)	Group 4 Typically developing infants as control (n = 50)	p	Post-hoc (group)
**Sex (female/male) (%/%)**		47/49 (49/51)	41/37 (52.6/47.4)	20/11 (64.5/35.5)	21/29 (42/58)	0.250^a^	
**Birth weight (grams)**	**Mean ± SD**	2501.04 ± 941.3	1609.67 ± 673.09	2405.48 ± 936.04	3131.5 ± 468.97	**<0.001** ^**b**^	1–2, 1–4, 2–3, 2–4, 3–4^d^
	**Range**	570–4560	570–3540	820–4100	2510–4000		
**Gestational age (weeks)**	**Mean ± SD**	35.01 ± 3.69	32.49 ± 3.32	34.26 ± 2.61	38.36 ± 1.24	**<0.001** ^**c**^	1–2, 1–4, 2–4, 3–4^e^
	**Range**	25–41	25–39	29–38	37–41		
**Recording age (weeks)**	**Mean ± SD**	13.4 ± 2.2	13.5 ± 2.3	14.0 ± 1.9	13.2 ± 1.7	0.207^c^	
	**Range**	10–19	9–19	11–18	11–18		
**Preterm birth, n (%)**		50 (52.1)	68 (87.2)	22 (71)	0	**<0.001** ^**a**^	1–2, 1–4, 2–4, 3–4^a^
**Large for gestational age, n (%)**		11 (11.5)	0	5 (16.1)	NA	**0.003** ^**a**^	1–2, 2–3^a^
**Small for gestational age, n (%)**		13 (13.5)	14 (17.9)	1 (3.2)	NA	0.130^a^	
**Intrauterine growth restriction, n (%)**		11 (11.5)	26 (33.3)	4 (12.9)	NA	**0.001** ^**a**^	1–2^a^
**Respiratory distress syndrome, n (%)**		17 (17.7)	16 (20.5)	4 (12.9)	NA	0.643^a^	
**Bronchopulmonary dysplasia, n (%)**		3 (3.1)	9 (11.5)	1 (3.2)	NA	0.057^a^	
**Patent ductus arteriosus, n (%)**		12 (12.5)	19 (24.4)	7 (22.6)	NA	0.111^a^	
**Necrotizing enterocolitis, n (%)**		4 (4.2)	1 (1.3)	1 (3.2)	NA	0.529^a^	
**Hyperbilirubinemia** ^**Φ**^ **, n (%)**		1 (1)	2 (2.6)	0	NA	0.576^a^	
**Periventricular leukomalacia, ≥ III, n (%)**		0	1 (1.3)	0	NA	0.441^a^	
**Intraventricular hemorrhage, ≥ III, n (%)**		1 (1)	1 (1.3)	0	NA	0.825^a^	
**Hypoxic-ischemic encephalopathy, ≥ II, n (%)**		0	0	0	NA	-	

^a^ Pearson Chi-Square test

^b^ One-way Anova test

^c^ Kruskal-Wallis test

^d^ Post hoc Tamhana T2 test

^e^ Mann Whitney-u test. Bold values indicate statistically significant at the p < 0.05 level.

^Φ^ Total serum bilirubin (TSB) value >12.9 mg/dl.

**Table 2:** Maternal characteristics.

	Group 1 Infants born to mothers with GDM (n = 96)	Group 2 Infants born to mothers with PE (n = 78)	Group 3 Infants born to mothers with GDM and PE (n = 31)	Group 4 Typically developing infants as control (n = 50)	p	Post-hoc (group)
**Maternal age (years)**						
**Mean ± SD**	32.37 ± 6.14	31.09 ± 5.81	33.39 ± 6.34	29.78 ± 5.82	**0.025** ^a^ *	-
**Range**	21–48	19–49	21–47	18–43		
**Maternal gravidity**						
**1, n (%)**	32 (33.30)	31 (39.7)	9 (29)	22 (44)	0.752^b^	-
**2–3, n (%)**	48 (50)	35 (44.9)	16 (51.6)	23 (46)		
**4+, n (%)**	16 (16.70)	12 (15.4)	6 (19.4)	5 (10)		
**Maternal parity**						
**Nulliparae, n (%)**	53 (55.2)	48 (61.5)	16 (51.6)	27 (54)	0.729^b^	-
**Multiparae, n (%)**	43 (44.8)	30 (38.5)	15 (48.4)	23 (46)		
**Multifetal pregnancies**						
**No, n (%)**	67 (45.6)	60 (76.9)	20 (64.5)	NA	0.366^b^	-
**Yes, n (%)**	29 (30.2)	18 (23.1)	11 (35.5)			
**Mode of delivery**						
**Normal vaginal delivery, n (%)**	13 (13.5)	4 (5.1)	4 (12.9)	21 (42)	**<0.001** ^b^	1–4, 2–4, 3–4^b^
**Cesarean, n (%)**	83 (86.5)	74 (94.9)	27 (87.1)	29 (58)		
**Maternal smoking during pregnancy**						
**Non-smoker, n (%)**	90 (93.8)	76 (97.4)	28 (90.3)	49 (98)	0.352^b^	-
**1–5 cigarettes/day, n (%)**	5 (5.2)	2 (2.6)	2 (6.5)	0		
**6–10 cigarettes/day, n (%)**	1 (1)	0	0	0		
**11 to 99 cigarettes/day, n (%)**	0	0	1 (3.2)	1 (2)		
**GDM treatment**						
**Diet, n (%)**	52 (54.2)	-	21 (67.7)	-	0.349^b^	-
**Diet and insulin, n (%)**	31 (32.3)	-	6 (19.4)	-		
**Missing Data, n (%)**	13 (13.5)	-	4 (12.9)	-		

^a^ One-way Anova test

^b^ Pearson Chi-Square test. Bold values indicate statistically significant at the p < 0.05 level.

* No p-value was less than 0.008 in the post-hoc analysis, only the p-value between group 3 and group 4 was 0.045.

Nulliparae women were defined as those with no previous live births before infants who included in this study, and multiparous women were those with second or more prior live births.

NA not applicable

### Bias

All databases were scanned by YT, EKB, and ZA, and infants born to mothers with GDM and/or PE referred, and video recorded between October 2021 and January 2022 were included in the study. After the video recordings of infants meeting the criteria were included, all video recordings were evaluated by two certified scorers (BNYL and AM).

### General movements assessment (GMA)

GMs are based on visual Gestalt perception and occur in age-specific patterns [30, 41]. When the infant’s age is between 3-and 5-months, they are called as fidgety movements which are small movements and variable acceleration of the neck, trunk, and limbs in all directions [30, 42]. Not only fidgety movements but also movement and postural patterns comprise the detailed GMA [30, 31]. Detailed GMA was evaluated from video recordings using the revised score sheet of the Motor Optimality Score for 3- to 5-Month-Old-Infants–Revised [31]. The revised score sheet comprises the following five subcategories: (i) temporal organization and quality of fidgety movements, (ii) observed movement patterns other than fidgety movements, (iii) age-adequate movement repertoire, (iv) postural patterns, and (v) movement character [30, 31]. The MOS is ranging from 28, indicating the best performance, to 5 [30, 31].

The video recordings were made with the partly dressed infant in the supine position during active wakefulness in the quiet room and without exposure to stimuli [43]. The video recordings were evaluated blindly and separately by two certified scorers (BNYL and AM) who were not familiar with the infants’ clinical history. In case of disagreement between the scorers (only 8 recordings of 205 infants: 3,9%), the video recordings were re-evaluated until consensus was reached. The intraclass correlation coefficient (ICC) of inter-observer reliability through a two-way mixed-effects model with consistency (average measures) for the MOS-R results was 0.979 (95% CI = 0.97–0.98) for two scorers in this study.

### Statistical analysis

The SPSS package for Macintosh, version 25.0 (SPSS Inc, Chicago, IL, USA), was performed for statistical analysis. To determine whether the variables were normally distributed or not, visual (histograms, probability plots) and analytical (Kolmogorov-Smirnov test) methods were used. We used the One-way ANOVA to compare continuous variables with a normal distribution (e.g., birth weight), and Levene’s test was used to assess the homogeneity of the variances. The Kruskal-Wallis test was for non-normally distributed data (e.g., gestational age, MOS). The Pearson chi-squared was used to compare the categorical variables (e.g., sex) between the groups. When an overall significance was observed, Tamhana T2 post-hoc test (for only birth weight) was performed following One-way ANOVA test, Mann Whitney-u test following Kruskal-Wallis test and for categorical variables was used the Pearson chi-squared test. P-values of less than 0.05 were considered statistically significant. Univariate logistic regression analyses were used to determine the odds ratios (OR) with 95% confidence intervals (CI).

## Results

**Fig 1:**
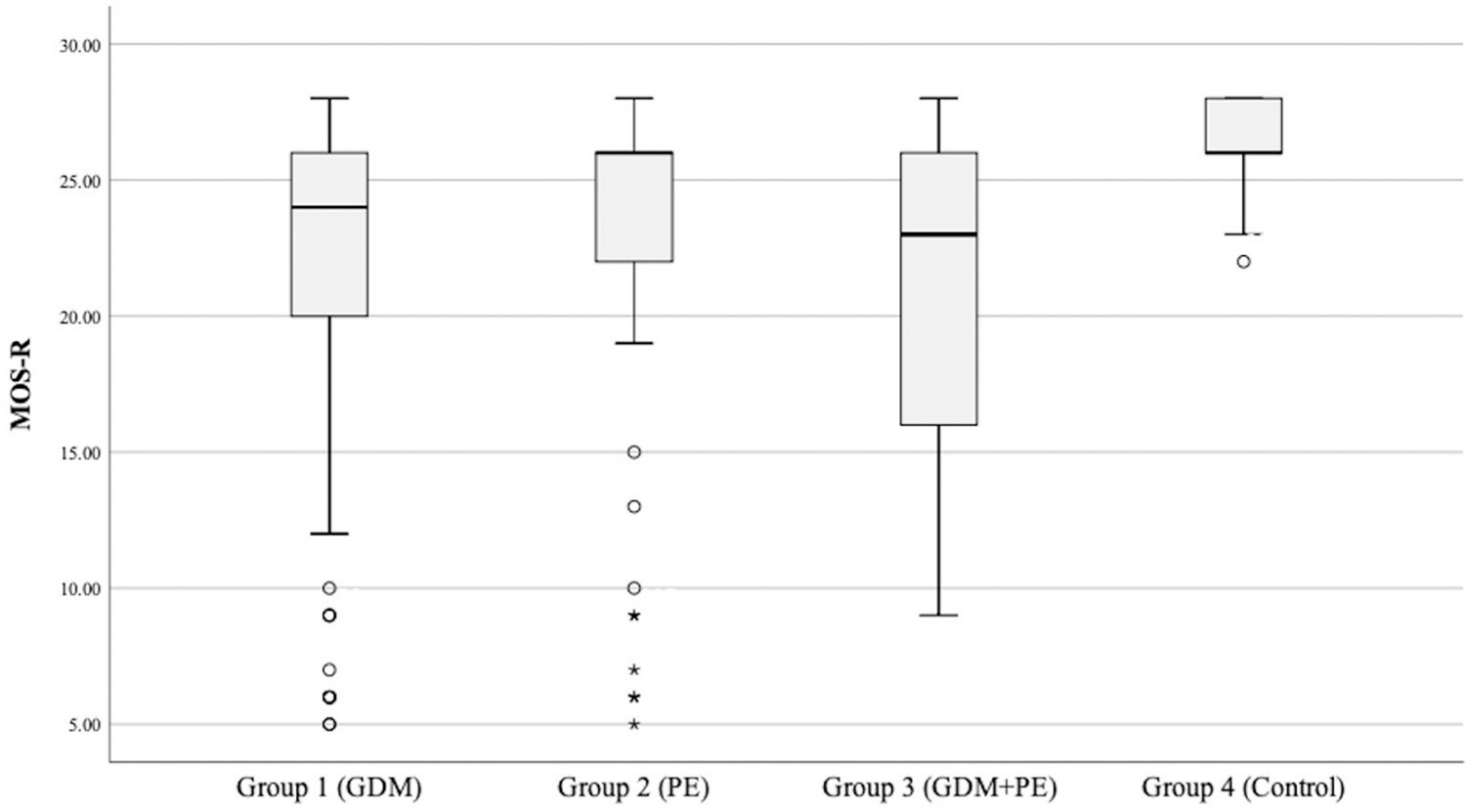
Motor optimality scores-revised of all groups.

There were no differences in the MOS-R results and fidgety movements among infants whose mothers had at least one of these risk factors (p > 0.05); in addition, infants with aberrant fidgety movements lad lower MOS-R in these three groups (p < 0.001) (Fig 1). Infants born to mothers with GDM, infants born to mothers with PE, and infants born to mothers with GDM and PE had lower MOS-R and more aberrant fidgety movements than typically developing infants (p < 0.001, p = 0.003, p < 0.001, respectively).

Infants born to mothers with GDM (*atypical age-adequate movement repertoire = 67*.*7%; atypical postural patterns = 38*.*5%*), infants born to mothers with PE (*atypical age-adequate movement repertoire = 47*.*4%; atypical postural patterns = 26*.*9%*), and infants born to mothers with GDM and PE (*atypical age-adequate movement repertoire = 70*.*9%; atypical postural patterns = 41*.*9%*) had a higher degree of atypical *age-adequate movement* repertoire (reduced or absent), and atypical postural patterns (N = A or N<A) than typically developing infants (p < 0.05). However, there were no differences in the quality of the observed movement patterns and movement character in all groups (p > 0.05).

The MOS-R and its subcategories’ results of all infants are shown in Table 3. The categorized MOS-R which is classified as severely reduced with a score from 5 to 8, moderately reduced with a score from 9 to 19, mildly reduced with a score from 20 to 24, and optimal with a score from 25 to 28, is given Table 3. Furthermore, the MOS-R of 17 infants (17.7%) in infants born to mothers with GDM, 10 infants (12.8%) in infants born to mothers with PE, and 6 infants (19.4%) in infants born to mothers with GDM and PE was 14 or below. In the control group, none of the infants had a score of 14 or below.

**Table 3:** MOS-R and its subcategories in all infants at 3–5 months post-term age.

MOS-R	Group 1 Infants born to mothers with GDM	Group 2 Infants born to mothers with PE	Group 3 Infants born to mothers with GDM and PE	Group 4 Typically developing infants as control	p	Post-hoc (group)
**Median**	24	26	23	26	**<0.001** ^**a**^	1–4, 2–4, 3–4^c^
**Min—Max (IQR)**	5–28 (20–26)	5–28 (22–26.5)	9–28 (16–26)	22–28 (26–28)		
**Optimal (25–28), (%)**	41 (42.7)	45 (57.7)	12 (38.7)	39 (78)	**<0.001** ^**b**^	1–4, 3–4^b^
**Mildly reduced (20–24), (%)**	32 (33.3)	22 (28.2)	10 (32.3)	11 (22)		
**Moderately reduced (9–19), (%)**	14 (14.6)	6 (7.7)	9 (29)	0		
**Severely reduced (5–8), (%)**	9 (9.4)	5 (6.4)	0	0		
**Fidgety movements**						
**Normal (%)**	78 (81.3)	68 (87.2)	23 (74.2)	50 (100)	**0.008** ^**b**^	1–4, 2–4, 3–4^b^
**Abnormal (%)**	2 (2.1)	0	2 (6.5)	0		
**Absent/Sporadic (%)**	16 (16.7)	10 (12.8)	6 (19.4)	0		
**Observed movement patterns**						
**N>A, (%)**	80 (83.3)	69 (88.5)	30 (96.8)	50 (100)	0.050^b^	**-**
**N = A, (%)**	5 (5.2)	4 (5.1)	0	0		
**N<A, (%)**	11 (11.5)	5 (6.4)	1 (3.2)	0		
**Age-adequate movement repertoire**						
**Age-adequate, (%)**	31 (32.3)	41 (52.6)	9 (29)	33 (66)	**<0.001** ^**b**^	1–4, 2–4, 3–4^b^
**Reduced, (%)**	22 (22.9)	9 (11.5)	9 (29)	15 (30)		
**Absent, (%)**	43 (44.8)	28 (35.9)	13 (41.9)	2 (4)		
**Observed postural patterns**						
**N>A, (%)**	59 (61.5)	57 (73.1)	18 (58.1)	48 (96)	**<0.001** ^**b**^	1–4, 2–4, 3–4^b^
**N = A, (%)**	13 (13.5)	7 (9)	8 (25.8)	2 (4)		
**N<A, (%)**	24 (25)	14 (17.9)	5 (16.1)	0		
**Movement character**						
**Smooth and fluent, (%)**	37 (38.5)	31 (39.7)	8 (25.8)	24 (48)	0.460^b^	-
**Abnormal, not CS, (%)**	56 (58.3)	46 (59)	22 (71)	26 (52)		
**CS, (%)**	3 (3.1)	1 (1.3)	1 (3.2)	0		

^a^ Kruskal-Wallis test

^b^ Pearson Chi-Square test

^c^ Mann Whitney-u test. IQR inter-quartile range. Bold values indicate statistically significant at the p < 0.05 level.

N>A, more normal than abnormal patterns; N = A, an equal number of normal and abnormal patterns; N<A, fewer normal than abnormal patterns; CS, cramped-synchronized movement character; MOS-R, Motor Optimality Score-Revised.

The odds ratio of having ≤ 24 in MOS-R was 4.76 (95% CI, 2.18–10.39, p ≤ 0.001) for infants born to mother with GDM, 2.74 (95% CI, 1.23–6.13, p = 0.013) for infants born to mother with PE, and 5.61 (95% CI, 2.10–15.03, p ≤ 0.001) for infants born to mother with GDM and PE (Table 4).

**Table 4:** Presented as odds ratios, including 95% confidence intervals, in optimal or suboptimal of the total MOS-R and its subcategories for GDM and/or PE vs control group.

	Fidgety movements Aberrant vs Normal	Observed movement patterns Suboptimal vs Optimal	Age-adequate movement repertoire Suboptimal vs Optimal	Observed postural patterns Suboptimal vs Optimal	Movement character Suboptimal vs Optimal	MOS-R ≤24 vs 25–28
	OR (95% CI)	OR (95% CI)	OR (95% CI)	OR (95% CI)	OR (95% CI)	OR (95% CI)
**Infants born to mothers with GDM vs Control group**	NA	NA	**4.07 (1.97–8.40)** **	**15.05 (3.45–65.66)** **	1.47 (0.74–2.94)	**4.76 (2.18–10.39)** **
**Infants born to mothers with PE vs Control group**	NA	NA	1.75 (0.84–3.65)	**8.84 (1.97–39.64)** **	1.40 (0.68–2.87)	**2.74 (1.23–6.13)** *
**Infants born to mothers with GDM and PE vs Control group**	NA	NA	**4.75 (1.80–12.54)** **	**17.33 (3.56–84.51)** **	2.65 (1.00–7.05)	**5.61 (2.10–15.03)** **

*p < 0.05

**p ≤ 0.001.

OR, odds ratio; CI, confidence interval; vs, versus, GDM gestational diabetes mellitus, MOS-R, motor optimality score-revised, NA, not applicable, 0 in one of the cells, PE pre-eclampsia.

## Discussion

In this study, we examined detailed GMA in infants born to mothers with GDM, with PE, and with both GDM and PE. Although there were other long-term studies on developmental outcomes in infants born to mothers with GDM or PE [16–19], our study was the first to performed detailed GMA and to compared together in these groups in the earliest period of life. The findings provided further insight into detailed GMA to identify infants at risk of neurodevelopmental disorder. Those infants whose mothers had at least one of these risk factors we mentioned had lower MOS-R, more aberrant fidgety movements, reduced age-adequate repertoire, and more abnormal postural patterns than typically developing infants; however, there were no differences in the movement character between all groups.

Einspieler et al. [31] indicated that in cases where neuroimaging is not available, a cramped-synchronized movement character, absent fidgety movements, and a MOS ≤ 14 are important signs that early intervention should be initiated. Our results showed that percentage of MOS score of less than 14 in our PE group was 12.8%, which was the lowest among the three groups, and all those infants also had absent fidgety movements. It might be important in those infants whose mothers had risk factors to determine whether they need early intervention in the clinic using detailed GMA.

Developing nervous system is critical for determining developmental functioning performance included cognitive, language, and motor domains [44, 45]. In the literature, studies reported that infants born to mothers with GDM or infants born to mothers with PE might have a risk of developmental problems [16–19] and might exhibit abnormality in different brain areas [21–25]. Kainer et al. [46] reported that type-I diabetes in the mother might affect the GMs in infants, which is in accordance with our results. Various complications, such as asphyxia, HIE, preterm birth, and IUGR, in the early period of life that might be caused by GDM and/or PE also present an increased risk factor for aberrant fidgety movements [39, 47–50]. On the other hand, a study conducted on infants with hyperbilirubinemia in which prematurity, perinatal asphyxia, and hemorrhage were excluded showed no differences in fidgety movements with the control group [40]. However, Vallamkonda et al. [51], recently reported that infants with hyperbilirubinemia had more aberrant fidgety movements than infants without hyperbilirubinemia. The present study also indicated that infants born to mothers with GDM and/or PE were also more likely to had aberrant fidgety movements than typically developing infants, but there was no difference in fidgety movements among infants whose mothers had at least one of these risk factors. This could be due to the various complications that we mentioned above, or there only being GDM and/or PE risk factors in infants’ mothers in our study sample; so those infants are prone to having neurological disorders.

In addition to detecting neurological disorders with fidgety movements, MOS is important to determine whether there is a risk for later developmental problems [36–38]. Recently, Salavati et al. [52] reported that a higher MOS-R and its subcategories were related to better performance for general cognition, attention, working memory, executive function, and motor function at 8 years of age in children born very preterm, which was consistent with another study in different developmental domains in children born preterm [53]. These findings were in line with other studies’ findings about risk factors may also be caused by GDM and/or PE [39, 40], which showed that infants who had various risk factors also had an increased risk of lower MOS (or MOS-R). However, Vallamkonda et al. [51], found no difference in MOS, unlike fidgety movements. This contrast in both fidgety movements and MOS results in these studies conducted on infants with hyperbilirubinemia [40, 51] might be due to the demographic characteristics of the, as Vallamkonda et al. said [51]. The difference in both fidgety movements and MOS results in our study suggests that in addition to a predisposition to neurological problems, these infants may have developmental problems without neurological problems. However, the relationship between MOS-R and later developmental outcomes needs to be investigated.

An interesting finding in our study was that there were no differences in movement character between all four groups, although control group had higher scores in all other MOS sub-categories than the other three groups. Recently Örtqvist et al. [54] reported that all infants born extremely preterm had an abnormal movement character but not cramped-synchronized. Fifty two percent of our infants in the control group had an abnormal movement character but not cramped-synchronized, and it was seen in the literature that its range in the control group was wide (between 32% and 73.3%) [39, 40, 54].

## Limitations

The main limitation of this study was its retrospective nature which caused the unequal sample size in our groups, and missing data in treatment during pregnancy. Other possible limitation was that we could not eliminate risk factors, such as preterm birth caused by GDM or PE; however, we reported all these risk factors according to groups. Finally, developmental outcomes at later ages were lacking to investigate the relationship between early spontaneous movements and developmental functioning problems.

## Conclusion

GDM and PE are common in pregnant women, and their presence and/or the comorbidities they cause might present a risk of abnormalities in the developing brain of infants. Infants born to mothers with GDM and/or PE had a higher degree of aberrant fidgety movements, abnormal postural patterns, and reduced repertoire than typically developing infants according to present study. In conclusion, our study findings suggest that early spontaneous movements assessment, the GMA, might help us to predict developmental outcomes of those infants and determine infants who need early intervention.
